# Effects of decitabine on allogeneic immune reactions of donor lymphocyte infusion via activation of dendritic cells

**DOI:** 10.1186/s40164-020-00178-y

**Published:** 2020-09-03

**Authors:** Yong-Rim Kwon, Hye Joung Kim, Min-Jung Sohn, Ji-Young Lim, Kyung-Shin Park, Seok Lee, Nack-Gyun Chung, Dae-Chul Jeong, Chang-Ki Min, Yoo-Jin Kim

**Affiliations:** 1 Laboratory of Hematological Disease and Immunology, Seoul, Republic of Korea; 2 Department of Clinical Pathology, Seoul St. Mary’s Hospital, Seoul, Republic of Korea; 3 Leukemia Research Institute, Seoul, Republic of Korea; 4 Seoul St. Mary’s Hematology Hospital, College of Medicine, The Catholic University of Korea, 222 Banpo-daero, Seocho-gu, Seoul, 06591 Republic of Korea; 5 Department of Pediatrics, Seoul St. Mary’s Hospital, College of Medicine, The Catholic University of Korea, Seoul, Republic of Korea

**Keywords:** Donor lymphocyte infusion, Decitabine, Graft-versus-host disease, Graft-versus-leukemia effect, Dendritic cells

## Abstract

**Background:**

Successful prevention of post-transplantation relapse after donor lymphocyte infusion (DLI) depends on its capability to mediate an effective graft-versus-leukemia (GVL) response while minimizing DLI-related toxicity, including graft-versus-host disease (GVHD).

**Methods:**

We assessed the effects of decitabine (DEC), a hypomethylating agent, upon allogeneic immune reaction in a murine model of DLI.

**Results:**

Significantly greater tumor growth retardation and survival prolongation occurred in mice administered with 1.0 mg/kg DEC for 5 days (DEC-1.0) than in control or DEC-0.1 mice. Upon prompt DEC and DLI co-administration, dendritic cells (DCs) were activated; DEC-1.0/DLI induced severe GVHD, and survival was significantly lower than with DLI alone or DEC-0.1/DLI treatments. IFN-γ and CD28 levels were higher in splenic DCs of DEC-1.0 mice than in those of control mice. Assessment of delayed DLI co-administration with DEC, when IFN-γ levels were normalized to control levels, revealed that DEC-1.0/DLI successfully facilitated tumor management without causing severe GVHD.

**Conclusions:**

Our results suggest that DEC primes allogeneic immune reactions of DLI via DC activation, and GVHD and GVL effects are separable through optimal DLI timing based on DEC-induced increase in IFN-γ expression levels.

## Background

Allogeneic hematopoietic stem cell transplantation (HSCT) is a curative treatment option for selected patients with hematological malignancies. The recent improvement in long-term survival after transplantation may result from a reduction in treatment-related mortality due to organ damage, infection, and severe acute graft-versus-host disease (GVHD) [1]; however, relapse remains the major cause of transplantation failure. Accordingly, various strategies to decrease post-transplantation relapse have been attempted, and incorporation of novel agents such as tyrosine kinase inhibitors (TKIs) or hypomethylating agents (HMAs) into pre- or post-HSCT settings is regarded as the most promising and feasible option. For example, a combination of TKIs and conventional chemotherapy in Philadelphia chromosome-positive acute lymphoblastic leukemia patients induced more pronounced molecular responses at the time of HSCT, and in turn significantly enhanced post-transplantation disease-free survival [2]. These beneficial effects of pre-HSCT TKI administration are primarily attributable to its direct antineoplastic cytotoxic effects. HMAs such as 5-azacitidine or 5-aza-2′-deoxycytidine (DEC) can induce direct antitumor toxicities similar to TKIs [3]. In addition, they may also affect tumors by modulating gene expression in key cellular regulatory pathways [4] and exerting their effects on various immune and immune-related cells [5–8]. In an HSCT setting, the administration of 5-azacitidine affected both acute and chronic GVHD [7, 9] and separation of graft-versus-leukemia (GVL) effects from GVHD was also suggested [7]. Overall, these data suggest that HMA administration modulates allogeneic immune reactions; however, further research on its role in cellular immunotherapies including donor lymphocyte infusion (DLI) is needed.

DLI is usually used to prevent imminent relapse or treat early-stage relapse, with the aim of inducing allogeneic immune responses against minimally residual leukemic cells; however, its overall success rate is relatively low due to limited GVL effects or unwanted adverse events, including severe GVHD or marrow aplasia [10, 11]. As limited antitumor effects of conventional DLI are associated with immune tolerance in cancer patients, various strategies have been attempted, including the use of combinations of conventional or novel chemotherapeutic agents [12, 13], induction of tumor antigens or costimulatory molecules [14, 15], or infusion of in vitro-generated leukemia-specific cytotoxic T lymphocytes [16, 17]. Mechanistically, successful tumor targeting by DLI depends on successful tumor antigen presentation and activation of antigen-presenting cells, such as dendritic cells (DCs). The potential of HMAs to disrupt immune tolerance may be due to their up-regulation of HLA, tumor antigens, and costimulatory molecules and induction of T cell responses against tumor antigens [8, 18–21]. More importantly, recent studies have supported the use of HMAs in combination with DLI, since HMAs induce generation or activation of DCs [5, 22], which are primarily involved in the development of immune reactions following allo-HSCT [23].

In the present study, we hypothesized that the administration of DEC activates host DCs and that this DEC priming may enhance the antitumor effects of subsequent DLI. To test this hypothesis, a mouse HSCT model mimicking early-stage relapse, wherein DLI is usually attempted clinically, was selected. Furthermore, to assess the adverse effects of DEC on GVHD, a mouse model wherein DLI typically fails to induce clinical signs of GVHD was used.

## Methods

### Mice and cell line

Female C57BL/6 (B6, H-2b) and B6D2F1 (F1, H-2b/d) mice were purchased from Orient Bio (Seongnam, Korea) and maintained in our facilities under pathogen-free conditions. Animal care and experiments were conducted in accordance with the guidelines of our institutional animal care and use committee. Murine mastocytoma P815 cells (H-2d) were used to verify GVHD and GVL effects based on previous studies [24] and were obtained from the American Type Culture Collection (ATCC, Manassas, VA, USA) and cultured in accordance with their instructions.

### Post-transplantation relapse mouse model

The early stage of post-transplantation relapse is characterized by a low tumor burden in mixed donor chimerism. Mixed chimeric status was modeled using a haploidentical (B6→F1) transplantation mouse model, receiving nonmyeloablative conditioning [25]. Briefly, an F-1 mouse was conditioned with 400 cGy of total body irradiation, and bone marrow cells at a density of 1 × 10^7^ cells were administered intravenously from B6 mice. The early-stage relapse with low tumor burden was modeled via subcutaneous inoculation of P815 cells at a density of 1 × 10^6^ cells 1 day before DEC administration to recipient mice.

### DEC administration with or without DLI

To investigate the effects of DEC on tumor growth, allogeneic immune reaction, and resultant mouse survival, DEC at variable doses was intraperitoneally administered once daily, and its effects were compared with those in the controls. In the transplantation setting, mice were divided into two groups following DEC administration: DLI and non-DLI. For DLI, spleen cells (2 × 10^7^) from B6 donors were resuspended in HBSS (Welgene, Daegu, Korea) supplemented with 2% FBS (GIBCO, Grand Island, NY, USA), washed twice, and injected into the tail vein of recipient mice at specified time points. Tumor growth was assessed every 3–4 days by measuring the largest perpendicular diameter with a caliper; thus, tumor sizes were recorded in terms of area (mm^3^). Survival was monitored daily, and the degree of clinical GVHD was assessed every 3–4 days based on weight loss, posture, the occurrence of diarrhea, and skin lesions, such as alopecia and dermatitis [26]. For histological analysis, hematoxylin and eosin staining was performed for the liver, skin, and intestinal tissues. DEC was purchased from Sigma-Aldrich (St. Louis, MO, USA).

### T and NK cell and cytokine assays

Frequencies of T cells (CD3 + CD4 + , CD3 + CD8 + , CD4 + CD25 + Foxp3 +) and NK cells (CD3-NK1.1 + B220 +) were analyzed using the following antibodies (BD, Franklin Lakes, NJ, USA): APC-cy7-conjugated anti-CD3 (Clone SK7), PE-conjugated anti-CD4 (Clone RPA-T4), PerCP-conjugated anti-CD8 (Clone RPA-T8), PerCP 5.5-conjugated anti-CD25 (Clone PC61), APC-conjugated-Foxp3 (Clone 259D/C7), PE-conjugated anti-NK1.1 (Clone PK136), and PerCP-conjugated anti-B220 (Clone RA3-6B2). The estimation and analysis of T cells, NK cells, and cytokines were performed using Fortessa (BD Biosciences, San Jose, CA, USA) and FlowJo software (Tree Star, Ashland, OR, USA). T-cell proliferative capacities were assessed via flow cytometric analysis of carboxyfluorescein diacetate succinimidyl ester (Invitrogen, Carlsbad, CA, USA) in the mixed lymphocyte reaction (MLR) with irradiated (13 Gy) splenic DCs (1 × 10^5^ cells) as stimulators. For cytokine analyses, serial serum samples were collected and analyzed via cytometric Bead Array flex kits (BD Biosciences) containing IFN-γ, TNF-α, IL-2, IL-6, IL-10, and IL-17A.

### DC preparation and phenotypical and functional analysis

Bone marrow-derived immature and mature DCs (imDCs and mDCs; im/mDCs) from F1 mice were generated in vitro, as described previously [27]. Briefly, imDCs were generated by culturing bone marrow cells in media containing granulocyte–macrophage colony-stimulating factor (GM-CSF; 10 ng/ml; Peprotech, Rocky Hill, NJ, USA) and IL-4 (10 ng/ml; Peprotech), while mDCs were generated by addition of lipopolysaccharide (1 μg/ml; Sigma-Aldrich) to the media after 6 days. The im/mDC phenotypes were analyzed via flow cytometry using CD40, CD54, CD80, CD86 (eBioscience Inc, San Diego, CA, USA), and major histocompatibility complex (MHC)-I and -II markers (BD). The bone marrow-derived DC function was analyzed via assessment of dextran (Sigma-Aldrich) uptake. Cytochalasin D was inhibited at a concentration of 10 μg/ml. Splenic DCs were isolated by applying CD11c-positive selection beads (Miltenyi Biotech Inc, CA, USA) to the spleen cells obtained from an F1 recipient mouse. The purified splenic CD11c + DCs were used for MLR and to analyze mRNA levels of 84 key genes involved in DC activation and maturation, using the RT^2^ Profiler PCR Array Kit (Qiagen, Germantown, MD, USA). Data were analyzed in accordance with the RT^2^ Profiler PCR Array Data Analysis Template, Version 3.3. Relative changes in gene expression based on DEC exposure were calculated using the ΔΔ*Ct* method, and a difference in gene expression of ≥ 1.5-fold at P < 0.05 was considered statistically significant. Real-time PCR was performed to quantify interferon-γ (IFN-γ) and CD28 in splenic DCs using the primers listed in Table 1.

**Table 1:** Primers used for Real-time PCR

Primer		Sequence (5′ → 3′)
CD28	F	TACTTCTGCAAAATTGAGTTCATC
	R	GGGGAGTCATGTTCAGTAG
*INF*-gamma	F	TGAACGCTACACACTGCATCTTGG
	R	CGACTCCTTTTCCGCTTCCTGAG
β-actin	F	AAGGCCCGTGAAAAGAT
	R	GTGGTACGACCAGAGGCATAC

### Statistical analyses

Data are presented as mean ± standard error of the mean. The Wilcoxon signed-rank test was performed to compare tumor sizes, body weights, T cell and NK cell numbers, and cytokine levels. Spearman’s rank test was performed for correlation analysis. Survival curves were plotted using the Kaplan–Meier method, and differences were evaluated using the log-rank test. P values < 0.05 were considered significant throughout.

## Results

### Selection of the optimal dose of DEC

We attempted to determine the appropriate dose of DEC for co-administration with DLI by assessing the antineoplastic effects in non-transplanted tumor-bearing mice and systemic toxicities in the transplantation model. First, F1 mice were subcutaneously inoculated with P815 (1 × 10^6^ cells), and a palpable mass was observed approximately 6 days after inoculation. On day 7, mice were divided into five groups, and daily doses of 0, 0.1, 0.5, 1.0, or 2.0 mg/kg body weight of DEC were administered intraperitoneally for 5 consecutive days, respectively. Tumor size reduction and survival prolongation after DEC treatment were observed at doses of 0.5 mg/kg or higher (Additional file 1: Figure S1a, b). DEC doses other than 2.0 mg/kg were then selected and administered on day 9 after transplantation to assess systemic toxicities in mice undergoing transplantation. No differences in body weight or survival of mice were observed among these three different doses (Additional file 1: Figure S1c, d), and hence, 0.1 and 1.0 mg/kg (DEC-0.1 and DEC-1.0, respectively) were selected to simplify subsequent experiments.

### Differential effects of DEC according to DLI

**Fig. 1:**
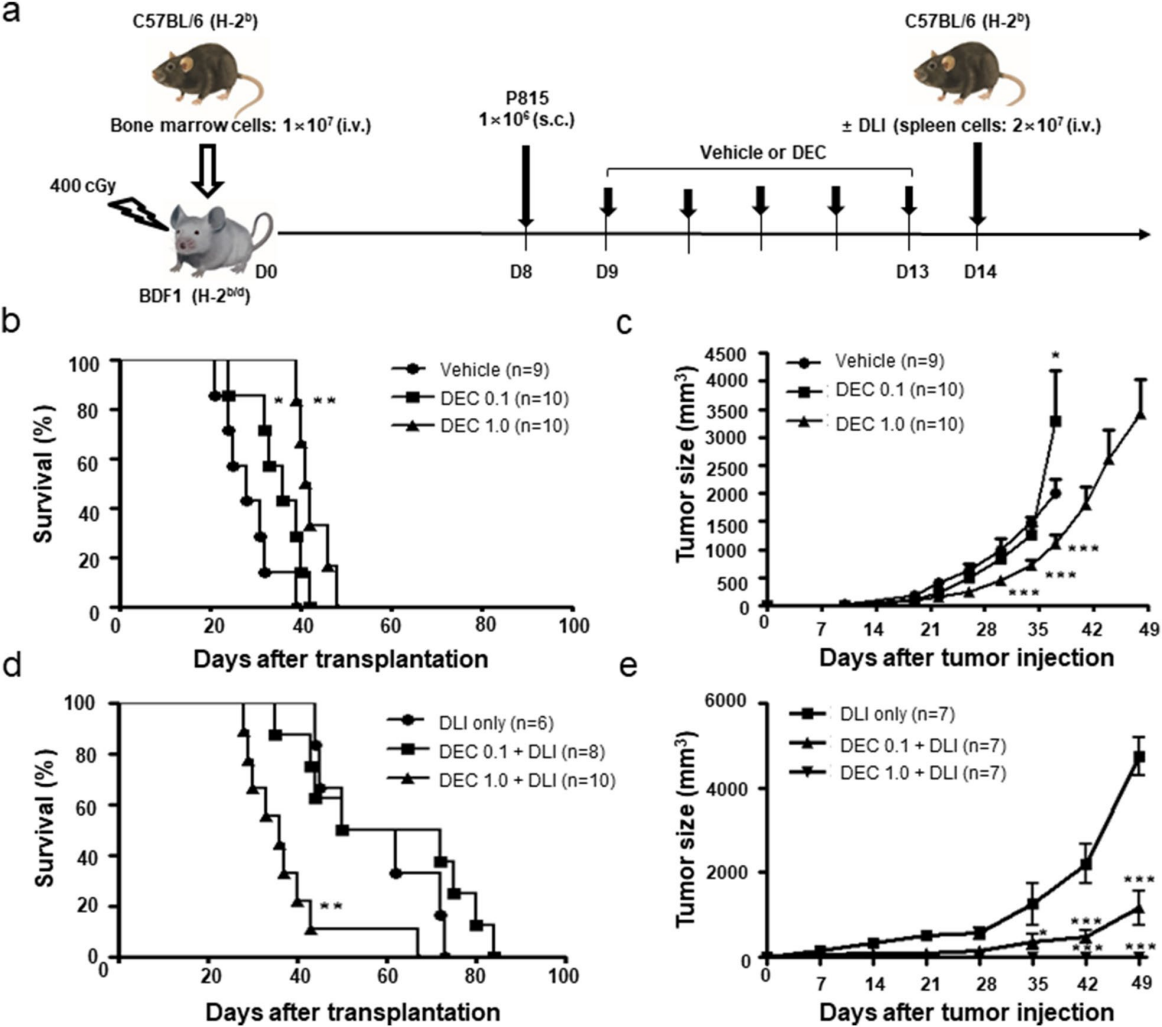
**a** Effect of decitabine (DEC) on tumor growth and survival in the absence or presence of donor lymphocyte infusion (DLI) in a post-transplantation early relapse mouse model. B6-derived bone marrow cells were transplanted to F1 mice after non-myeloablative conditioning, and DEC was injected following the inoculation of P815 cells. Survival and tumor size were assessed in the **b, c** absence or **d, e** presence of DLI. The experiment was conducted in 3 times.*P < 0.05, **P < 0.01, ***P < 0.001 for control vs. DEC-treated group

**Fig. 2:**
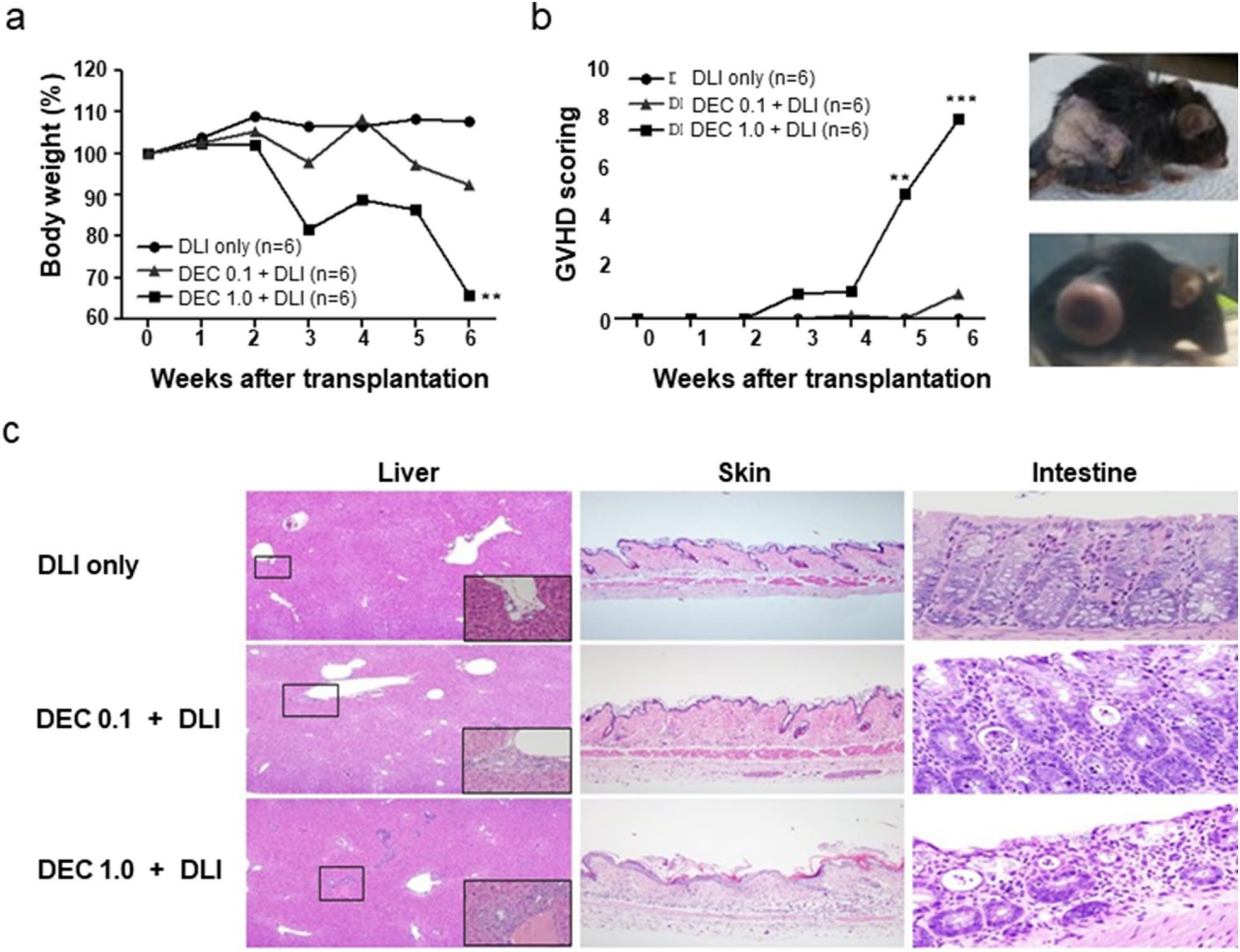
Alloimmune reaction after combinatory treatment with decitabine (DEC) and donor lymphocyte infusion (DLI). DEC-1.0 enhanced allogeneic immune reaction of DLI, which induced severe graft-versus-host disease (GVHD) as evidenced by **a** body weight loss, **b** clinical GVHD score, and **c** histology in the liver, skin, and intestine while inducing successful graft-versus-tumor effects. The experiment was conducted in 3 times.**P < 0.01, ***P < 0.001 for control vs. DEC-treated group

Figure 1a shows the experimental design of DEC administration with or without DLI in a mouse model of early-stage relapse in a mixed chimeric state. In the absence of DLI, DEC-1.0 group showed significantly greater survival prolongation and tumor growth retardation than the control and DEC-0.1 groups (Fig. 1b, c). When DLI was combined with DEC, mice in the DEC-1.0/DLI group showed significantly shorter survival but greater tumor reduction compared with the DLI only or DEC-0.1/DLI groups (Fig. 1d, e). Similar to the DLI only group, delayed tumor growth was observed in the DEC-0.1/DLI group, which resulted in prolonged survival. Mice administered DEC-1.0/DLI showed a progressive decrease in body weight (Fig. 2a) and higher clinical GVHD score compared with mice administered DLI only or DEC-0.1/DLI (Fig. 2b). Histological analyses also revealed that the pathological findings were compatible with those of GVHD and were most prominent in the DEC-1.0/DLI group (Fig. 2c). These data suggest that DEC-1.0 can enhance the allogeneic immune reaction of DLI, thus inducing severe GVHD and concomitant GVL effects.

### Changes in immune cells and cytokines following DEC administration

**Fig. 3:**
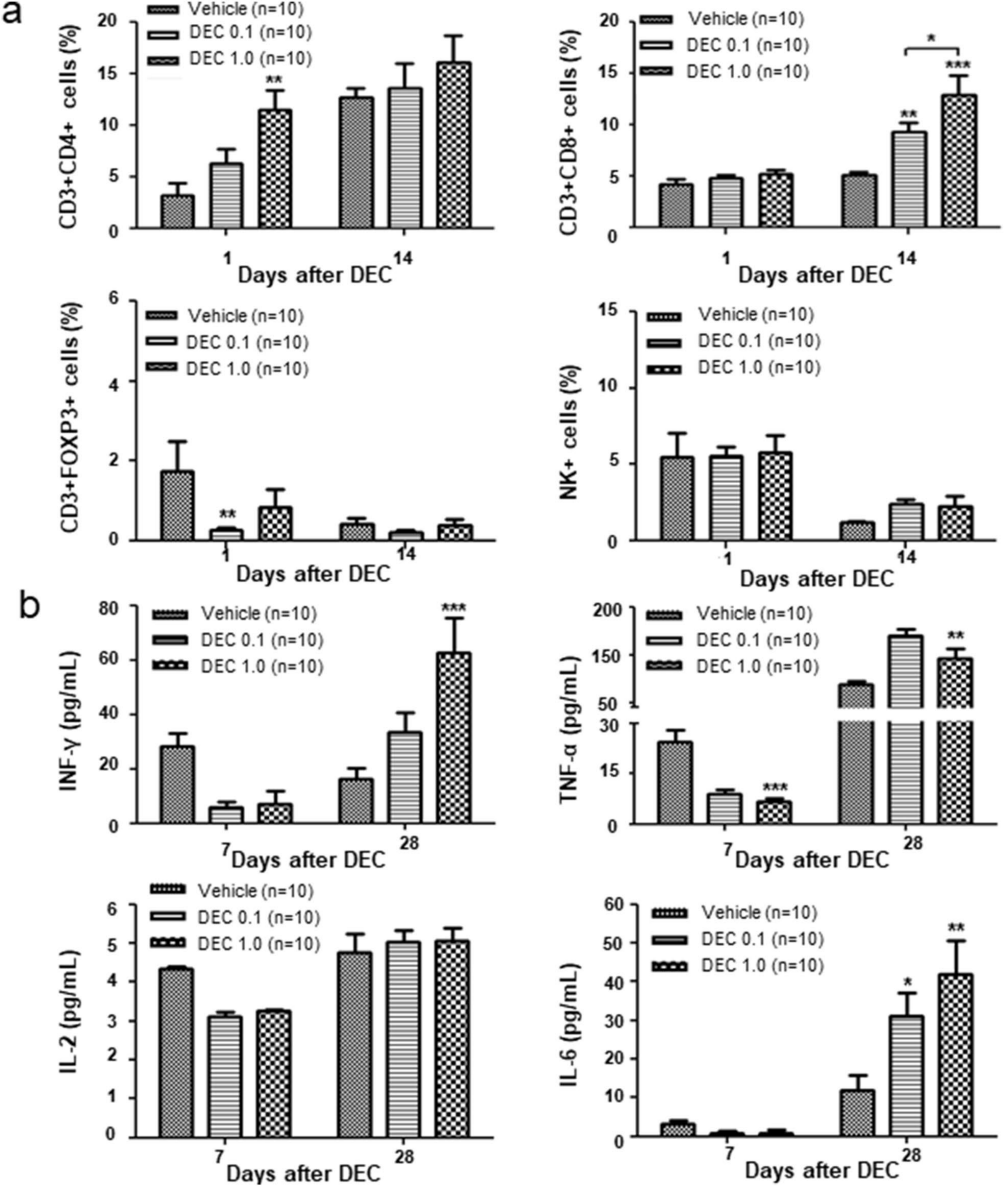
**a** Changes in CD3 + CD4 + , CD3 + CD8 + , and CD4 + CD25 + FOXP3 + T cells and NK cells and **b** various cytokines after decitabine administration with or without donor lymphocyte infusion. The experiment was conducted in 3 times.*P < 0.05, **P < 0.01, ***P < 0.001 for control vs. DEC-treated group

To assess the effect of DEC on immune cells in mice undergoing transplantation, peripheral blood samples were analyzed 1 day after DEC treatment and 14 days after DEC and DLI co-administration. DEC treatment alone significantly increased the frequency of CD3 + CD4 + T cells in mice on day 1, while decreasing that of regulatory T-cells; however, differences in the frequency of immune cells between the groups were not statistically significant. Two weeks after DEC and DLI co-administration, when severe GVHD occurred, we observed a significantly high frequency of CD3 + CD8 + T cells. Nonetheless, frequencies of NK cells were not affected by DEC treatment only or with DLI co-administration (Fig. 3a). Serum cytokine levels decreased 7 days after DEC administration; however, IFN-γ level was significantly increased for approximately 28 days in mice administered DEC-1.0/DLI and exhibiting GVHD (Fig. 3b).

### Phenotypical and functional changes in DCs by DEC administration

**Fig. 4:**
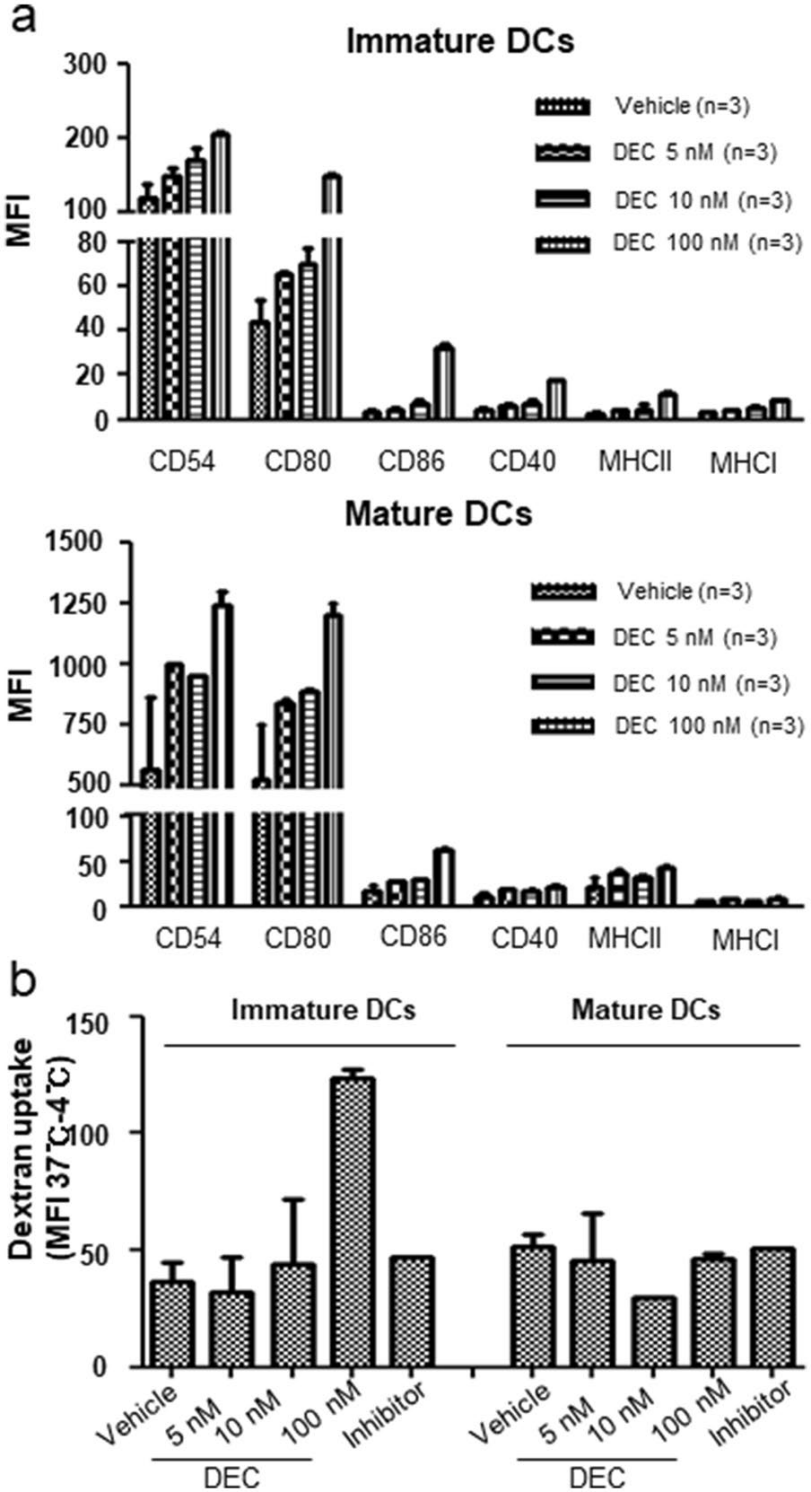
Decitabine (DEC)-induced phenotypical and functional changes of dendritic cells (DCs). **a** After marrow-derived im/mDCs were treated with various doses of DEC in vitro, changes in DC surface markers and **b** functional changes in dextran uptake were assessed

**Fig. 5:**
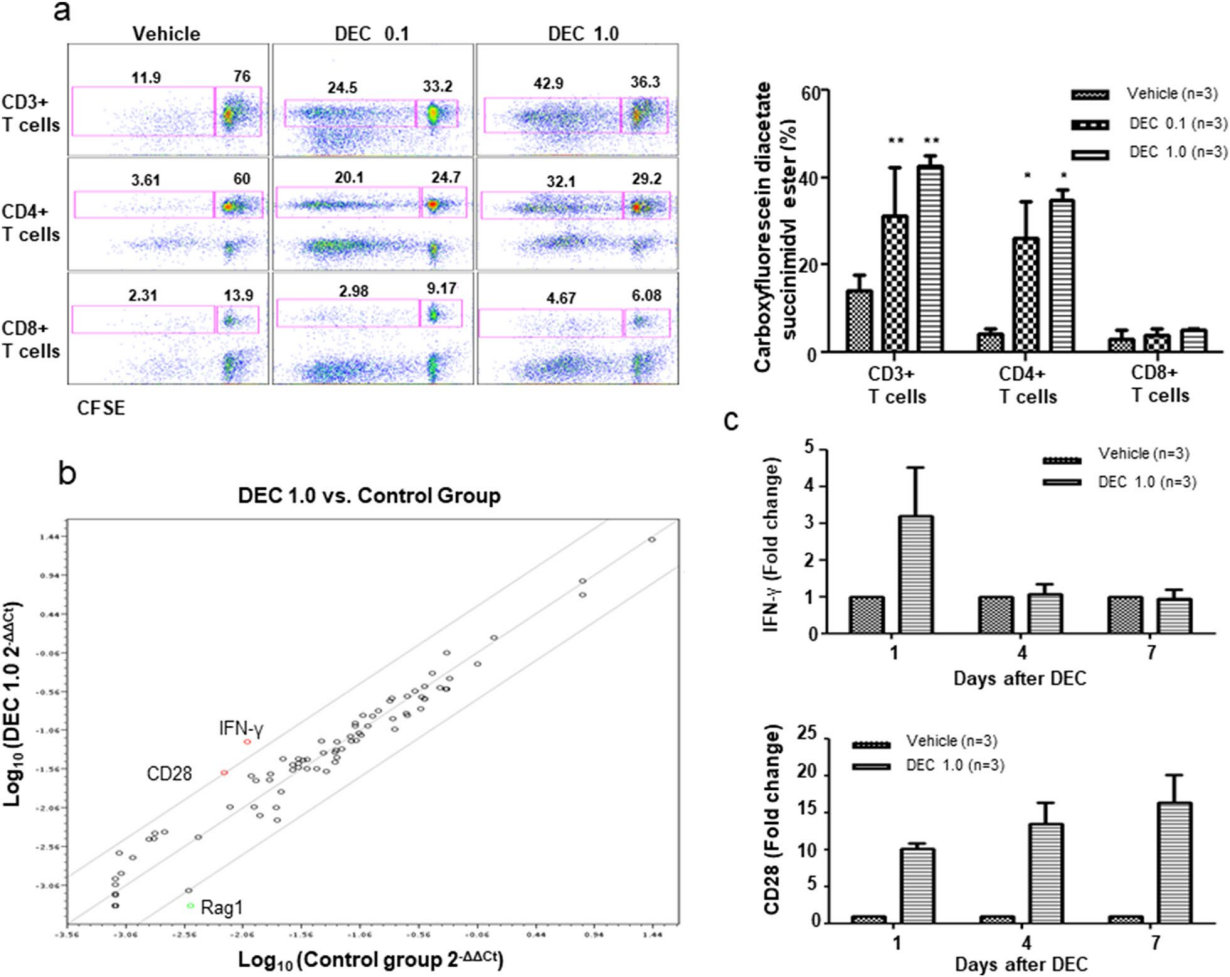
Effects of decitabine (DEC)-treated dendritic cells (DCs) on T cell proliferation and associated molecules. **a** Splenic DCs isolated from recipient mice treated with or without DEC were co-cultured with donor T cells, and proliferation of CD3 + , CD3 + CD4 + , and CD3 + CD8 + T cells was assessed via the carboxyfluorescein diacetate succinimidyl ester method. **b** Changes in the expression of molecules following DEC administration assessed using a gene kit of 84 genes known to be involved in DC function, and **c** the dynamics of IFN-γ and CD28 expression over time analyzed by real-time polymerase chain reaction

Regarding the role of host DCs in acute GVHD [23], we speculated whether DEC-induced GVHD aggravation was associated with DC activation. First, we investigated the phenotypical changes in marrow-derived DCs, which were induced in vitro with DEC treatment. The expression of CD54, CD80, and CD86 was upregulated in both im/mDCs and that of CD40 and MHC class II was upregulated in imDCs; dextran uptake by imDCs was also enhanced by DEC administration. These findings suggest that DEC treatment functionally modified DCs (Fig. 4a, b). We then selected CD11c + splenic DCs from recipient mice on day 1 after DEC administration to induce an MLR with donor T cells. Carboxyfluorescein diacetate succinimidyl ester assay showed that CD3 + and CD3 + CD4 + T-cell proliferation induced upon exposure to splenic DCs from mice administered DEC-0.1 and DEC-1.0 was significantly higher than that induced by vehicle (Fig. 5a), suggesting that DEC induced DC activation in a dose-dependent manner.

### IFN-γ dynamics of DCs and influence on GVHD and GVL effects

**Fig. 6:**
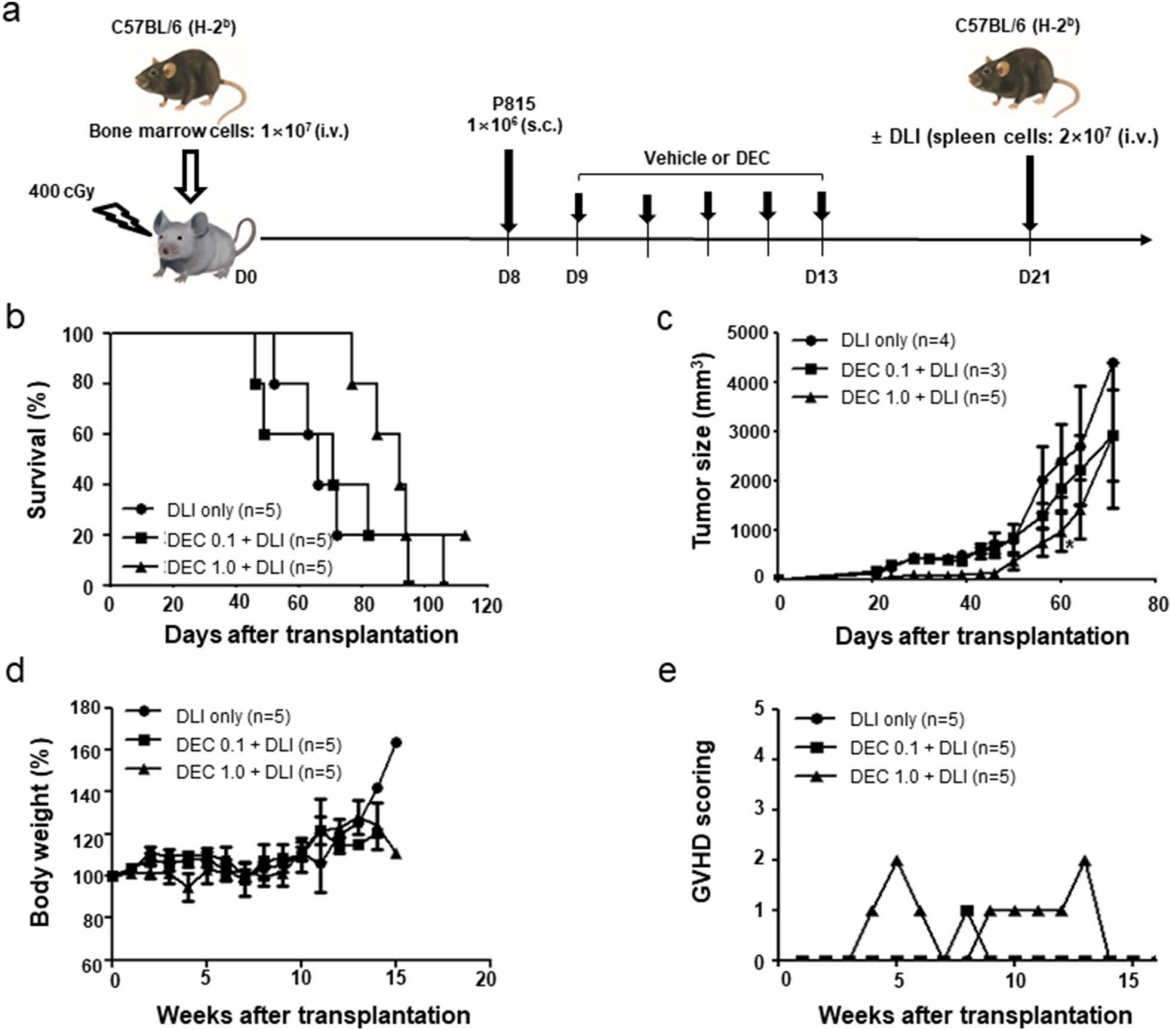
**a** After injecting C57BL6 mouse-derived bone marrow cells into irradiated BDF1 mice, P815 cells were transplanted subcutaneously 8 days later. Decitabine was injected on days 1–5 after tumor transplantation, and donor lymphocyte infusion (DLI) was performed the day after injection. Changes observed in **b** survival, **c** tumor size, **d** body weight, and **e** GVHD scoring depending on the delay in DLIs. The experiment was conducted in 3 times. *P < 0.05 for control vs. DEC-treated group

Next, we assessed the DEC-mediated molecular changes in DCs using a commercial kit containing 84 genes associated with DC function. CD11c + splenic cells from day 1 after drug exposure were used, and upregulation of IFN-γ and CD28 expression was observed in splenic DCs from mice administered DEC-1.0 (Fig. 5b). We also analyzed IFN-γ and CD28 mRNA expression levels using real-time PCR and showed that their levels were significantly higher in splenic cells on day 1 after exposure to DEC-1.0 (Fig. 5c). However, the dynamics of IFN-γ and CD28 expression differed. Upon DEC exposure, IFN-γ expression levels increased and were normalized to control levels over time. Conversely, CD28 levels were persistently higher than the control values. Next, to assess the association between GVHD aggravation and IFN-γ levels in splenic DCs post-administration of DEC-1.0 and DLI, DLI was delayed until 8 days after DEC administration, when IFN-γ levels normalized to control values (Fig. 6a). Greater tumor size reduction and more prolonged survival were observed when DEC-1.0 was administered with DLI than in those groups where mice received only DLI or DEC-0.1 and DLI (Fig. 6b, c). We found that delayed DLI failed to induce severe GVHD, as evidenced by changes in body weight (Fig. 6d) and the clinical GVHD score (Fig. 6e).

## Discussion

Although HMAs are currently being used to prevent or treat post-transplant relapse either by administering this drug alone [28–30] or in combination with DLI [31–33]; to the best of our knowledge, the mechanism-based roles of HMAs in DLI and their effects on GVHD/GVL have not been analyzed. The purposes of this study were to determine whether pretreatment with DEC enhances allogeneic immune reactions following DLI, whether DCs are associated with altered GVHD/GVL effects, and to evaluate the possibility of modulating immune reaction following DEC and DLI co-administration through analysis of the DEC-induced DC activation mechanism. The mouse bone marrow transplantation model was chosen because GVHD typically does not occur after DLI in this model, resulting in more accurate and reliable information regarding the influence of DEC on GVHD induction.

Inadequate antigen presentation is an immune escape mechanism employed by malignant cells [34], and disrupting this immune tolerance is a central step for successful anti-cancer immunotherapy. Accordingly, DCs are crucial for DLI success in terms of their roles in T-cell priming and the development of an immune reaction [23]. Maturation of DCs into effective antigen-presenting cells is a complex process involving epigenetic cell fate control and functional modification of DCs by epigenetic drugs; in fact, decreased antigen-presenting activity of DCs by histone deacetylase inhibitors has been observed [35, 36]. Meanwhile, HMAs have been suggested to stimulate DC activity [22, 36, 37].

In this study, the immune stimulatory influence of DEC upon DCs was first evidenced through the maturation and activation of DCs following in vitro drug treatment, characterized by higher expression levels of class II MHC molecules and co-stimulatory molecules such as CD80, CD86, and CD54, compared with those in the controls. Functional enhancement of DEC-treated DCs was then confirmed by dextran uptake and MLR; compared with the control, higher proliferation of CD4 + T cells was observed when splenic DCs from mice administered with DEC were used. In line with these observations, the T-cell proliferation pattern was indicative of the effects of DEC on DCs and subsequent immune reactions. High induction of CD4 + T cells soon after DEC administration, especially following DEC-1.0 treatment, was followed by a significant increase in CD8 + T cell levels in DEC/DLI groups at later stages than in the DLI-only group. Regarding the critical importance of CD4 + T cell in the induction and maintenance of tumor antigen-specific CD8 + T cells [38], we assumed that low antigen specificity of conventional polyclonal DLI could be overcome by DEC. Overall, these results suggest that enhanced DC function by DEC (relative to DLI-only group) is associated with significant tumor reduction and severe GVHD in mice administered with DEC-1.0/DLI. Finally, analysis of gene panels related to antigen presentation showed significant upregulation of CD28 and IFN-γ expression in CD11c + splenic DCs from mice administered with DEC, coinciding with the role of IFN-γ in DC activation [22].

We also investigated the mechanisms underlying DEC-induced aggravation of allogeneic immune reactions other than DC activation by assessing the changes in cytokines and immune cells other than CD4 + and CD8 + T cells. Consistent with previous studies using 5-azacitidine [7, 39], our results showed that DEC downregulated the expression of inflammatory cytokines. Contrary to observations in previous studies where 5′-azacitidine was used [40–42], the regulatory T cell frequency was decreased by DEC in this study. Of note, differences in HMA type, immune cell analysis timing, and administration schedules could also affect the differential influences of HMAs on regulatory T cells. Although decrements in regulatory T cells might have contributed, DEC-induced DC activation played a dominant role in enhanced allogeneic immune reactions following combined treatment with DEC and DLI.

Our study showed that DEC induced DC activation and potentiated DLI-induced GVL effects as well as unwanted severe GVHD, emphasizing the need for strategies to separate these effects. Based on our observation that the IFN-γ levels increased by DEC returned to control levels over time (as seen in humans) [43], delayed DLI administration was performed on day 8 post-DEC administration. This resulted in reduced GVHD severity even when combined with a higher dose of DEC. However, we observed better anti-tumor activity and longer survival in mice receiving DEC and DLI than in those administered with DLI alone. These results suggest that bone marrow cells are more sensitive to GVHD than other target organs, and separation of GVHD and GVL effects is possible. Different cell number thresholds exist for GVHD and GVL effects following conventional DLI [44, 45]. Similarly, our results suggest that different thresholds may exist for DC activation status. In addition to assessing the optimum time interval between DEC and DLI, we also tested alternative approaches for the infusion of different cell numbers 1 day after DEC administration. However, infusion of a ten-fold lower number of lymphocytes failed to prevent severe GVHD (data not shown), which further suggested that DC activation plays a greater role in the induction of DEC-induced DC activation-mediated GVHD than just the number of lymphocytes. Together, these findings suggest the possible use of DEC and DLI combination as a therapeutic tool, warranting further research geared toward clinical application. Based on these observations, we are planning to investigate the effects of IFN-γ or CD28 expression levels of DCs on the occurrence of GVHD or GVL effects following DLI in patients treated with HMA and DLI. Moreover, the optimal DEC dosing schedule for appropriate DC activation, the interval between DEC and DLI, and DC activation markers other than IFN-γ should be investigated.

## Conclusions

In conclusion, DEC-induced activation of DCs was shown to facilitate the eradication of remnant tumor cells via GVL effects, and the activation status of DCs could be optimized by the time interval between DEC and DLI to help separate GVL effects from unwanted GVHD.

## Supplementary information

**Additional file 1: Figure S1.** Optimal decitabine (DEC) dose selection for co-administration with donor lymphocyte infusion. Effects of DEC on tumor growth and survival in a non-transplant setting: **a** tumor size and **b** survival. Systemic toxicities of DEC in the haploidentical (B6→F1) transplantation mouse model: **c** survival and **d** body weight. The experiment was conducted in 2 times.

## Data Availability

Not applicable.

## Abbreviations

DLI: Donor lymphocyte infusion
GVL: Graft-versus-leukemia
DCs: Dendritic cells
DEC: Decitabine
GVHD: Graft-versus-host disease
HSCT: Hematopoietic stem cell transplantation
TKIs: Tyrosine kinase inhibitors
HMAs: Hypomethylating agents

## References

[1] Gooley TA, Chien JW, Pergam SA (2010). Reduced mortality after allogeneic hematopoietic-cell transplantation. N Engl J Med.

[2] Lee S, Kim DW, Cho BS (2012). Impact of minimal residual disease kinetics during imatinib-based treatment on transplantation outcome in Philadelphia chromosome-positive acute lymphoblastic leukemia. Leukemia.

[3] Yahng SA, Kim M, Kim TM (2017). Better transplant outcome with pre-transplant marrow response after hypomethylating treatment in higher-risk MDS with excess blasts. Oncotarget..

[4] Tsai HC, Li H, Van Neste L (2012). Transient low doses of DNA-demethylating agents exert durable antitumor effects on hematological and epithelial tumor cells. Cancer Cell.

[5] Daurkin HC, Eruslanov E, Vieweg J (2010). Generation of antigen-presenting cells from tumor-infiltrated CD11b myeloid cells with DNA demethylating agent 5-aza-2′-deoxycytidine. Cancer Immunol Immunother.

[6] Schmiedel BJ, Arélin V, Gruenebach F (2011). Azacytidine impairs NK cell reactivity while decitabine augments NK cell responsiveness toward stimulation. Int J Cancer.

[7] Sánchez-Abarca LI, Gutierrez-Cosio S, Santamaría C (2010). Immunomodulatory effect of 5-azacytidine (5-azaC): potential role in the transplantation setting. Blood.

[8] Goodyear O, Agathanggelou A, Novitzky-Basso I (2010). Induction of a CD8 + T-cell response to the MAGE cancer testis antigen by combined treatment with azacitidine and sodium valproate in patients with acute myeloid leukemia and myelodysplasia. Blood.

[9] Fransolet G, Ehx G, Somja J (2016). Azacytidine mitigates experimental sclerodermic chronic graft-versus-host disease. J Hematol Oncol..

[10] Schmid M, Labopin M, Nagler A (2007). Donor lymphocyte infusion in the treatment of first hematological relapse after allogeneic stem-cell transplantation in adults with acute myeloid leukemia: a retrospective risk factors analysis and comparison with other strategies by the EBMT Acute Leukemia Working Party. J Clin Oncol.

[11] Roddie C, Peggs KS (2011). Donor lymphocyte infusion following allogeneic hematopoietic stem cell transplantation. Expert Opin Biol Ther..

[12] Levine JE, Braun T, Penza SL (2002). Prospective trial of chemotherapy and donor leukocyte infusions for relapse of advanced myeloid malignancies after allogeneic stem-cell transplantation. J Clin Oncol.

[13] Shimoni A, Kröger N, Zander AR (2003). Imatinib mesylate (STI571) in preparation for allogeneic hematopoietic stem cell transplantation and donor lymphocyte infusions in patients with Philadelphia-positive acute leukemias. Leukemia.

[14] Levine BL, Bernstein WB, Connors M (1997). Effects of CD28 costimulation on long-term proliferation of CD4 + T cells in the absence of exogenous feeder cells. J Immunol..

[15] Laport GG, Levine BL, Stadtmauer EA (2003). Adoptive transfer of costimulated T cells induces lymphocytosis in patients with relapsed/refractory non-Hodgkin lymphoma following CD34 + -selected hematopoietic cell transplantation. Blood.

[16] Falkenburg JH, Wafelman AR, Joosten P (1999). Complete remission of accelerated phase chronic myeloid leukemia by treatment with leukemia-reactive cytotoxic T lymphocytes. Blood.

[17] Kim YJ, Cho SG, Lee S (2010). Potential role of adoptively transferred allogeneic WT1-specific CD4 + and CD8 + T lymphocytes for the sustained remission of refractory AML. Bone Marrow Transplant.

[18] Coral S, Sigalotti L, Gasparollo A (1999). Prolonged upregulation of the expression of HLA class I antigens and costimulatory molecules on melanoma cells treated with 5-aza-2′-deoxycytidine (5-AZA-CdR). J Immunother.

[19] Sigalotti L, Altomonte M, Colizzi F (2003). 5-Aza-2′-deoxycytidine (decitabine) treatment of hematopoietic malignancies: a multimechanism therapeutic approach?. Blood.

[20] Almstedt M, Blagitko-Dorfs N, Duque-Afonso J (2010). The DNA demethylating agent 5-aza-2′-deoxycytidine induces expression of NY-ESO-1 and other cancer/testis antigens in myeloid leukemia cells. Leuk Res.

[21] Atanackovic D, Luetkens T, Kloth B (2011). Cancer-testis antigen expression and its epigenetic modulation in acute myeloid leukemia. Am J Hematol.

[22] Frikeche J, Clavert A, Delaunay J (2011). Impact of the hypomethylating agent 5-azacytidine on dendritic cells function. Exp Hematol.

[23] Duffner UA, Maeda Y, Cooke KR (2004). Host dendritic cells alone are sufficient to initiate acute graft-versus-host disease. J Immunol..

[24] Lim JY, Choi MS, Youn H (2011). The influence of pretransplantation conditioning on graft-vs.-leukemia effect in mice. Exp Hematol..

[25] Choi MS, Lim JY, Cho BS (2011). The role of regulatory T cells during the attenuation of graft-versus-leukemia activity following donor leukocyte infusion in mice. Leuk Res.

[26] Cooke KR, Kobzik L, Martin TR (1996). An experimental model of idiopathic pneumonia syndrome after bone marrow transplantation: I. The roles of minor H antigens and endotoxin. Blood..

[27] Inaba K, Inaba M, Romani N (1992). Generation of large numbers of dendritic cells from mouse bone marrow cultures supplemented with granulocyte/macrophage colony-stimulating factor. J Exp Med.

[28] de Lima M, Giralt S, Thall PF (2010). Maintenance therapy with low-dose azacitidine after allogeneic hematopoietic stem cell transplantation for recurrent acute myelogenous leukemia or myelodysplastic syndrome: a dose and schedule finding study. Cancer.

[29] Han S, Kim YJ, Lee J (2015). Model-based adaptive phase I trial design of post-transplant decitabine maintenance in myelodysplastic syndrome. J Hematol Oncol..

[30] Platzbecker U, Middeke JM, Sockel K (2018). Measurable residual disease-guided treatment with azacitidine to prevent haematological relapse in patients with myelodysplastic syndrome and acute myeloid leukaemia (RELAZA2): an open-label, multicentre, phase 2 trial. Lacet Oncol..

[31] Schroeder T, Czibere A, Platzbecker U (2013). Azacitidine and donor lymphocyte infusions as first salvage therapy for relapse of AML or MDS after allogeneic stem cell transplantation. Leukemia.

[32] Schroeder T, Rachlis E, Bug G (2015). Treatment of acute myeloid leukemia or myelodysplastic syndrome relapse after allogeneic stem cell transplantation with azacitidine and donor lymphocyte infusions–a retrospective multicenter analysis from the German Cooperative Transplant Study Group. Biol Blood Marrow Transplant.

[33] Platzbecker U, Wermke M, Radke J (2012). Azacitidine for treatment of imminent relapse in MDS or AML patients after allogeneic HSCT: results of the RELAZA trial. Leukemia.

[34] Cardoso AA, Schultze JL, Boussiotis VA (1996). Pre-B acute lymphoblastic leukemia cells may induce T-cell anergy to alloantigen. Blood.

[35] Reddy P, Sun Y, Toubai T (2008). Histone deacetylase inhibition modulates indoleamine 2,3-dioxygenase-dependent DC functions and regulates experimental graft-versus-host disease in mice. J Clin Invest..

[36] Stepanek I, Indrova M, Bieblova J (2011). Effects of 5-azacytidine and trichostatin A on dendritic cell maturation. J Biol Regul Homeost Agents.

[37] Triozzi PL, Aldrich W, Achberger S (2012). Differential effects of low-dose decitabine on immune effector and suppressor responses in melanoma-bearing mice. Cancer Immunol Immunother.

[38] Chakraverty R, Eom HS, Sachs J (2006). Host MHC class II + antigen-presenting cells and CD4 cells are required for CD8-mediated graft-versus-leukemia responses following delayed donor leukocyte infusions. Blood.

[39] Choi J, Ritchey J, Prior JL (2010). In vivo administration of hypomethylating agents mitigate graft-versus-host disease without sacrificing graft-versus-leukemia. Blood.

[40] Goodyear OC, Dennis M, Jilani NY (2012). Azacitidine augments expansion of regulatory T cells after allogeneic stem cell transplantation in patients with acute myeloid leukemia (AML). Blood.

[41] Schroeder T, Fröbel J, Cadeddu RP (2013). Salvage therapy with azacitidine increases regulatory T cells in peripheral blood of patients with AML or MDS and early relapse after allogeneic blood stem cell transplantation. Leukemia.

[42] Chan MW, Chang CB, Tung CH (2014). Low-dose 5-aza-2′-deoxycytidine pretreatment inhibits experimental autoimmune encephalomyelitis by induction of regulatory T cells. Mol Med.

[43] Lau J, Sartor M, Bradstock KF (2007). Activated circulating dendritic cells after hematopoietic stem cell transplantation predict acute graft-versus-host disease. Transplantation.

[44] Johnson BD, Truitt RL (1995). Delayed infusion of immunocompetent donor cells after bone marrow transplantation breaks graft-host tolerance allows for persistent antileukemic reactivity without severe graft-versus-host disease. Blood.

[45] Mackinnon S, Papadopoulos EB, Carabasi MH (1995). Adoptive immunotherapy evaluating escalating doses of donor leukocytes for relapse of chronic myeloid leukemia after bone marrow transplantation: separation of graft-versus-leukemia responses from graft-versus-host disease. Blood.

