# Supplementary figures and images for: Effects of decitabine on allogeneic immune reactions of donor lymphocyte infusion via activation of dendritic cells
Source: Exp Hematol Oncol. 2020 Sep 3;9:22. doi: 10.1186/s40164-020-00178-y (PMC7470611; doi:10.1186/s40164-020-00178-y)

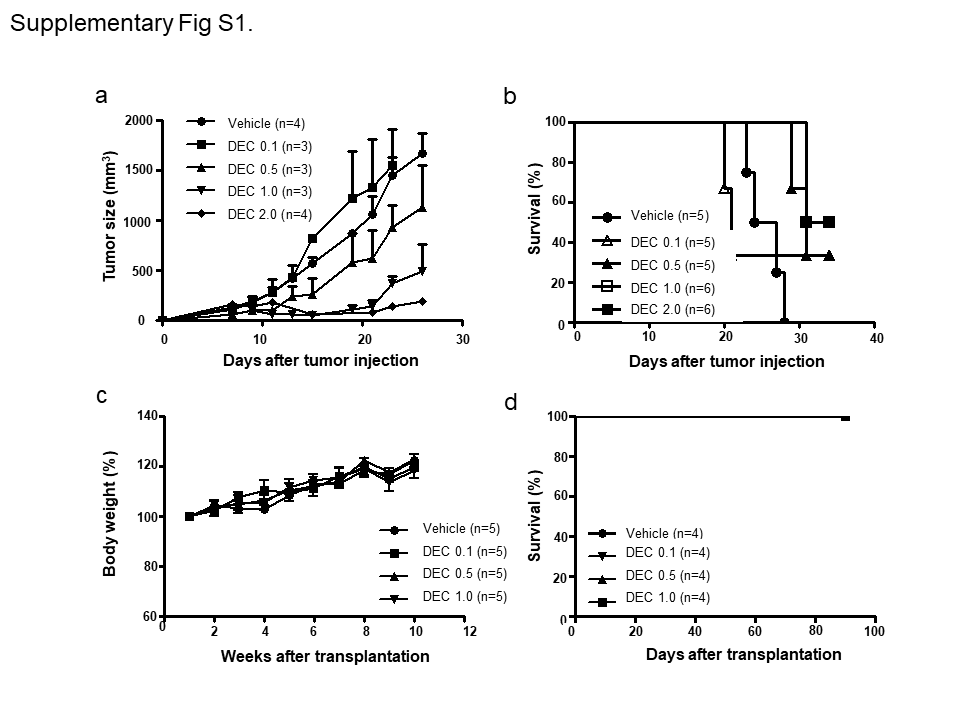

Supplement: Supplementary file 1 — Additional file 1: Figure S1. Optimal decitabine (DEC) dose selection for co-administration with donor lymphocyte infusion. Effects of DEC on tumor growth and survival in a non-transplant setting: a tumor size and b survival. Systemic toxicities of DEC in the haploidentical (B6→F1) transplantation mouse model: c survival and d body weight. The experiment was conducted in 2 times. [file 40164_2020_178_MOESM1_ESM.tif]
